# Characterization and Cytotoxic Assessment of Ballistic Aerosol Particulates for Tungsten Alloy Penetrators into Steel Target Plates

**DOI:** 10.3390/ijerph7093313

**Published:** 2010-08-26

**Authors:** Brenda I. Machado, Lawrence E. Murr, Raquel M. Suro, Sara M. Gaytan, Diana A. Ramirez, Kristine M. Garza, Brian E. Schuster

**Affiliations:** 1 Department of Metallurgical and Materials Engineering, The University of Texas at El Paso, El Paso, TX 79968, USA; E-Mails: lemurr@utep.edu (L.E.M.); smgaytan@miners.utep.edu (S.M.G.); daramirez2@miners.utep.edu (D.A.R.); 2 Department of Biological Sciences, The University of Texas at El Paso, El Paso, TX 79968, USA; E-Mails: rmsuro@miners.utep.edu (R.M.S.); kmgarza@utep.edu (K.M.G.); 3 U.S. Army Research Laboratory, Weapons and Materials Research Directorate (RDRL-WML-H), USA; E-Mail: bschuster@arl.army.mil (B.E.S.)

**Keywords:** tungsten alloys, nanoparticulates, cytotoxic assays, scanning and transmission electron microscopy, aerosol, ballistic penetration

## Abstract

The nature and constituents of ballistic aerosol created by kinetic energy penetrator rods of tungsten heavy alloys (W-Fe-Ni and W-Fe-Co) perforating steel target plates was characterized by scanning and transmission electron microscopy. These aerosol regimes, which can occur in closed, armored military vehicle penetration, are of concern for potential health effects, especially as a consequence of being inhaled. In a controlled volume containing 10 equispaced steel target plates, particulates were systematically collected onto special filters. Filter collections were examined by scanning and transmission electron microscopy (SEM and TEM) which included energy-dispersive (X-ray) spectrometry (EDS). Dark-field TEM identified a significant nanoparticle concentration while EDS in the SEM identified the propensity of mass fraction particulates to consist of Fe and FeO, representing target erosion and formation of an accumulating debris field. Direct exposure of human epithelial cells (A549), a model for lung tissue, to particulates (especially nanoparticulates) collected on individual filters demonstrated induction of rapid and global cell death to the extent that production of inflammatory cytokines was entirely inhibited. These observations along with comparisons of a wide range of other nanoparticulate species exhibiting cell death in A549 culture may suggest severe human toxicity potential for inhaled ballistic aerosol, but the complexity of the aerosol (particulate) mix has not yet allowed any particular chemical composition to be identified.

## 1. Introduction

In regards to very small fragments and fragment aerosols, there have been few systematic observations of associated particulate chemistries, size distributions or cytotoxic responses, especially pertinent to respiratory inflammatory responses or more serious respiratory health effect indicators; although recent work by Gold *et al.* [1] has examined aerosols inside an armored vehicle penetrated by a kinetic energy tungsten heavy alloy (KE WHA) penetrator. In addition, Guillmette *et al.* [2] have also discussed the health risk for depleted uranium (DU) aerosols. It is now well established that ultra-fine or nanoparticulate materials characteristic of a wide compositional range and particulate morphologies exhibit respiratory inflammatory and cytotoxic effects for a range of human lung cell types [3–10].

The present study is concerned with the systematic collection of aerosol particulates associated with ballistic WHA rod penetration into rolled homogeneous armor (RHA) or related steel armor or armor plate sequences in a containment vessel. This research is also concerned with the characterization of these collected particulates using scanning and transmission electron microscopy; including the analysis of particulate chemistries or elemental compositions utilizing energy dispersive (X-ray) spectrometry (EDS). Finally, filter-collected aerosol particulates were exposed to human epithelial (lung) cells in culture to assess their inflammatory and related respiratory health effects.

## 2. Experimental Procedures

### 2.1. Materials and Ballistic Testing

**Figure 1.**
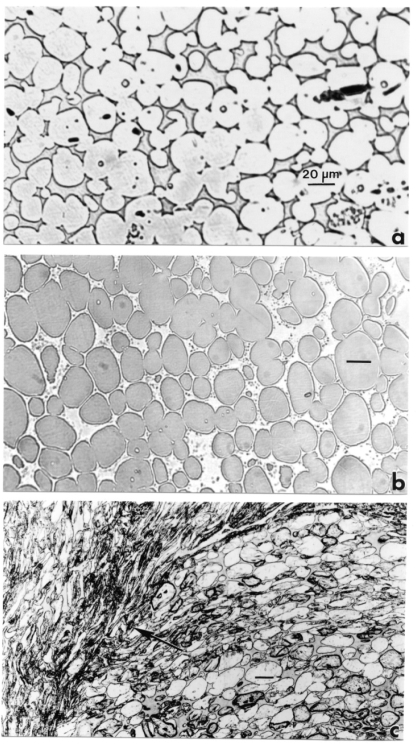
(a) WHA KE penetrator section views. **(a)** W-Ni-Fe penetrator. **(b)** W-Ni-Co penetrator. The spherical or near spherical particles in **(a)** and **(b)** are W in the corresponding alloy matrix.

Sub-scale WHA penetrators were fired into an array of mild steel plates which were encapsulated in a steel containment vessel. The penetrators were hemispherical-nose, 65 g rods with a length to diameter ratio of 20. The WHA rods consisted of either 91% (by weight) W, 5.6% Ni, 1.4% (WA #1) or 92% W, 6% Ni, 3% Co (WA #2) or and were fabricated using liquid phase sintering [11]. In Figure 1(a) and (b), we show the typical microstructures of WA #1 and #2 (respectively) consisting of pure tungsten particles surround by the matrix phase.

The target array consisted of 10 each of 6.25 mm thick mild steel plates spaced 12.5 mm apart backed by a thick RHA block to capture the residual penetrator. The penetrators were fired from a 26 mm smooth bore cannon outfitted with a 37 mm breach. The launch package consisted of a polypropulux laboratory sabot and obdurator with a steel pusher plate. The penetrators (and entire launch package) were fired at a velocity of ∼1.2 km/s using approximately 170 g of type M2 propellant. The approximate composition of the M2 propellant as reported by Roth and Watchtell is shown in Table 1 [12]. In all 12 tests, the penetrators completely perforated all of the thin mild steel plates and were caught in the thick RHA block.

**Figure 2.**
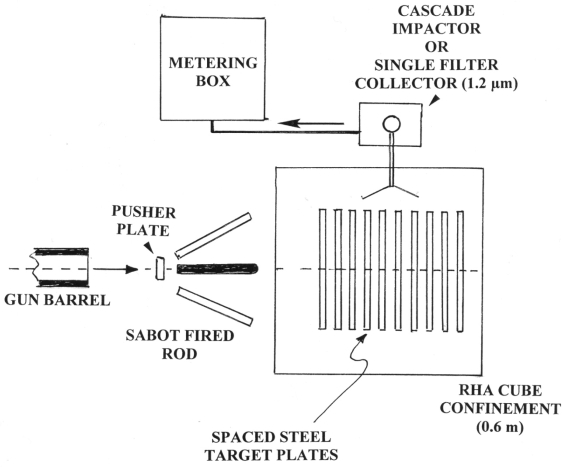
Ballistic aerosol particulate generation and collection. The sabot-fired KE rod penetrates 10 mild steel target plates in an RHA cube confinement. A cascade impactor or single-filter collector collects particulates from the debris fields in separate penetration events.

The containment vessel was an RHA cube with a wall thickness of ∼25 mm, with an internal open volume that was approximately 60 cm on a side. This vessel had a removable top which allowed for placement of the target array. The vessel had two ports: a ∼10 cm diameter port through which the WHA penetrators were fired and a smaller ∼6 mm port to allow for aerosol sampling (discussed below). This containment vessel is not a closed system as the larger porter is open during the ballistic test and throughout the sampling period. In Figure 2, we show a representative schematic of the ballistic test set-up including the penetrator launch package, mild steel target array and containment vessel.

### 2.2. Aerosol Collection and Analysis

Immediately following the ballistic test, aerosol samples were collected using either single-stage filters or by an eight-stage cascade impactor, under non-sterile conditions. The debris (particulate aerosol) generated by the rod impact, penetration and perforation as well as the residual propellant gases were sampled for ∼15 minutes through the respective collection media with an airflow rate of 2 L/min.

The single stage filters were SKC model 225–321A mixed cellulose ester (MCE) filters with a diameter of 25 mm and a pore-size of 0.8 μm. The eight stage cascade impactor utilized Mylar substrates with cut-points ranging from 0.5 to 21 μm and with a final backup filter with a 0.25 μm pore size. The Mylar substrates were sprayed with Dow Corning 316 silicone release lubricant ∼16h before initial weighing to provide an adhesive surface for the airborne particles. The total particulate concentrations at each cut point were determined from gravimetric analysis. Each substrate was retained for subsequent analysis in their SKC and Millipore containers and samples for the relative concentrations of W, Ni, Fe and Co. Analysis was conducted following National Institute for Occupational Safety and Health (NIOSH) method 7074 which included cellulose ester filter collection, acid digestion, and analysis by inductively coupled plasma optical emission spectroscopy (ICP-OES). EPA method 200.7 was used for metal analysis.

### 2.3. Aerosol Particulate Characterization

Filter collection samples were halved or quartered and utilized for direct particulate collection observation and analysis by scanning electron microscopy (SEM) and energy-dispersive (X-ray) spectrometry (EDS). The SEM was a field emission SEM (Hitachi S4800) operated at 20 kV accelerating potential to assure adequate excitation potential for EDS. Filter sections were also scraped onto silicon monoxide/formvar-coated 200 mesh, 3 mm copper grids and a second grid placed on top to form a sandwich for direct observation in the transmission electron microscope (TEM). The single (MCE) filter collections were more suitable for scraping particles onto TEM grids or onto sticky carbon supports for SEM. TEM image analysis was performed either in a Hitachi H-8000 electron microscope operated at 200 kV or a high-resolution TEM (Hitachi H-9500) operating at 300 kV and fitted with a goniometer-tilt stage. Selected-area electron diffraction (SAED) was utilized to observe the degree of crystallinity of the collected particulates and to allow for selective dark-field (DF) imaging utilizing specific diffraction spots. The high-resolution TEM (Hitachi H-9500) operating at 300 kV was also utilized especially for ultra-fine particle (nanoparticle) analysis. It was fitted with an EDAX-EDS system which allowed for nano-probe (point) analysis and scanned-area analysis.

### 2.4. *In Vitro* Cytotoxicity Assays for Particulate Collections

In this study we utilized direct exposure cytotoxicity assays for filter-collected ballistic particulates as described previously by Soto *et al.* [8]. Assays were performed for six different assay groups as summarized in Table 2. Direct exposure assays were performed using particulate specimens collected using the single-stage filters (SSF) associated with ballistic impacts of WA #1 and WA #2 into the steel target array. There was no measurement of particle weight on the filters. Untreated cells (or “media” only cells) served as the negative control; cells exposed to a filter on which nothing had been collected served as the blank control.

These assays measure *in vitro* cell viability or cell death using colorimetric or optical densitometry analysis along with cytokine enzyme linked immunosorbant assay (ELISA) studies which measure the up-regulation and release of interleukins by the cells. The type of ELISA kit used was a BD Biosciences. In recent ELISA studies, we utilized the immortalized A549 human epithelial lung cell line which provides an effective *in vitro* lung cell model and has been widely adopted as a human lung cell model. The cell line was obtained by the American Tissue Culture Collection (ATCC) from lung carcinomatous tissue from a 58 year old Caucasian male. The A549 cells were cultured in 12-well plates at 0.25 × 10^6^ cells per well with F-12 Ham’s media supplemented with 10% Fetal Bovine Serum (FBS) and 5% penicillin/streptomycin (PS) for several hours to allow the cells to adhere. The cells were then exposed for 48hs (standard time for acute exposures) with 1/4 of the indicated filter with the collection side facing towards the monolayer of cells. Following the exposure period, the filters were removed; the cells were harvested, and were then transferred into a 96-well flat bottom plate to assess viability via the MTS Assay. This colorimetric assay assesses relative viability as a function of color, which is directly proportional to the amount of cells available to convert the substrate into a color product. CellTiter 96 Aqueous One Solution Reagent (Promega), which contains the tetrazolium compound 3-(4, 5-dimethylthiazol-2-yl)-5-(3-carboxymethoxyphenyl)-2-(4-sulfophenyl)-2H-tetrazolium, inner salt (MTS), was added to each well. The plate was then incubated for 2 h at 37 °C in a humidified, 5% CO_2_ atmosphere. Finally, the absorbance was recorded at 490 nm using a 96-well plate spectrophotometer reader. The plate was a VersaMax Tunable Microplate Reader of Molecular Devices. Relative viability results were obtained by 1way ANOVA with Bonferroni’s Multiple Comparison Test.

Interleukin (IL)-6 and IL-8 secretion by A549 cells was measured using a commercial human IL-6 and IL-8 enzyme-linked immunosorbent assay (ELISA) kit (Biosource Human IL-6/IL-8 CytoSet). Supernatants were obtained 48hs after exposure to the different filters and were stored at −20 °C until subjected to ELISA analysis following the manufacturer’s protocol. The ELISA plates were coated 12 to 18 h at 4 °C with the capture antibody (Anti-Human IL-6 or IL-8). The plates were then blocked at room temperature with assay buffer for 1 h to prevent non-specific antigen binding. Next, the standards (Recombinant Human IL-6 or IL-8) and samples were added in duplicate (2 wells per treatment), immediately followed by the addition of the working detection antibody (Anti-Human IL-6 or IL-8 Biotin) and incubated for 2 h at room temperature. Subsequently, the plates were washed and the working streptavidin-horseradish peroxidase (HRP) solution was added to each well for 30 min. The enzyme substrate solution tetramethylbenzidine (TMB) was added for color development and finally, the enzyme reaction was stopped by the addition of the stop solution containing hydrosulfuric acid (H_2_SO_4_). Absorbance was measured at 450 nm using a 96-well plate spectrophotometer reader.

## 3. Results and Discussion

### 3.1. Aerosol Particulate Size Distribution and Composition

In Tables 3 and 4, we should the typical sampling data associated with particulate specimens captured using the cascade impactors for the WHA rod impact regime. As noted previously, these specimens were sampled to determine the relative fractions of W, Ni, Fe and Co using ICP-OES while the total particulate weights (and concentrations) were determined using gravimetric methods. The “Total Particulate Concentrations” presumably include a large fraction of undetermined particulate including organic compounds and residual compounds resulting from combustion of the M2 propellant

At all particulate sizes, Fe is the predominant constituent of all the metals samples. Throughout the respirable zone (including stages 3 through 8 of the cascade impactor), Fe-containing particulates are a factor of roughly 25 times the combine Ni and W concentrations. Analysis of the backup filter shows a smaller relative ratio of Fe to W, however the metallic constituents make up a much smaller relative fraction; for example in table 3, the total particulate concentration of 35 mg/m^3^ includes only ∼7.5 mg/m^3^ from W, Ni and Fe.

### 3.2. Particulate Characterization by SEM and TEM

**Figure 3.**
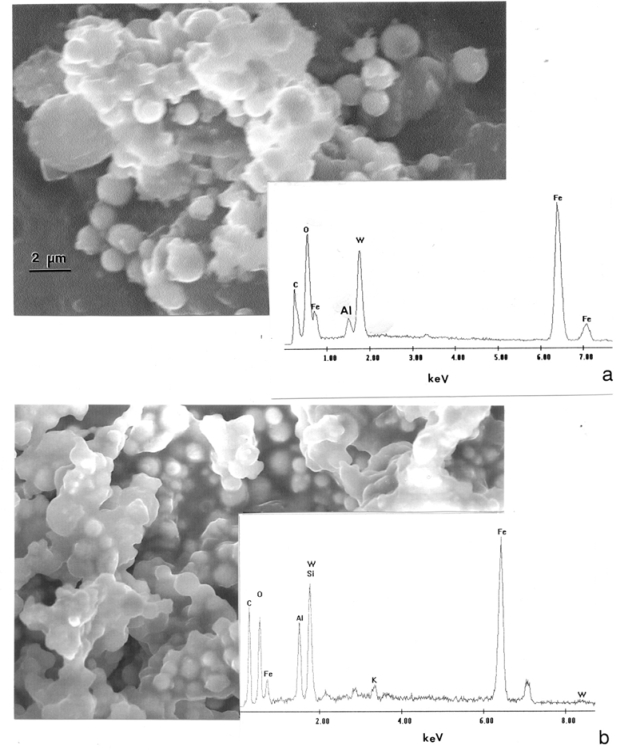
Stage 6 (Tables 1 and 2) cascade impactor filter samples observed in the FESEM. (a) WA #1 WHA penetrator event. (b) WA #2 WHA penetrator event. The corresponding energy-dispersive X-ray spectra (EDS) are inserted.

Figure 3 shows typical stage 6 (∼2 μm cut point) particulate (cascade impactor) collections observed directly in the SEM. Figure 3(a) illustrates particle aggregates for WA #1 projectile firing. Consistent with the particle analyses shown in Table 3, the dominant element shown in the EDS insert is Fe, with some observable W. There is no detectable Ni. The Fe found in the SEM/EDS analysis includes contributions from both the WHA matrix as well as from the mild steel target array; the latter is believed to provide the most significant contribution. It is not possible to separate the contribution from Fe found originally in the WHA from that in the target array. It was also observed that most of the spherical particles, ranging from 1 to 2 μm in Figure 3(a), were Fe. Figure 3(b) illustrates, similar particle aggregates for a penetration rod of WA #2 corresponding to Table 4. Similar to Figure 3(a), the EDS insert illustrates a propensity of target Fe, and there is no Ni or Co detected.

**Figure 4.**
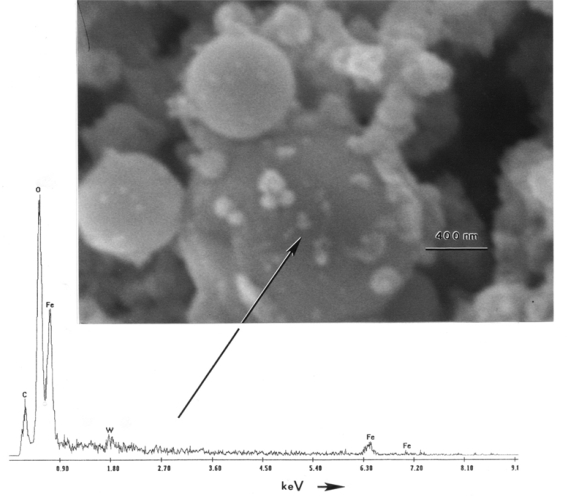
Single filter sample observed with the FESEM for a W-NiFe WHA penetrator event. The corresponding energy-dispersive X-ray spectrum for the large, spherical iron particle (arrow) is included.

Figure 4 illustrates an aggregate of particles observed for a typical MCE (single-filter) collection in the SEM. The propensity of particles ranging from 0.5 to 2 μm are spherical Fe particles. The EDS illustrates a similar composition (as found throughout) dominated by Fe, with some W. The EDS insert in Figure 4 also shows significant oxygen suggesting that many particles may be oxides: Fe-O (Fe_2_O_3_) as suggested in a previous study by Gold [13].

**Figure 5.**
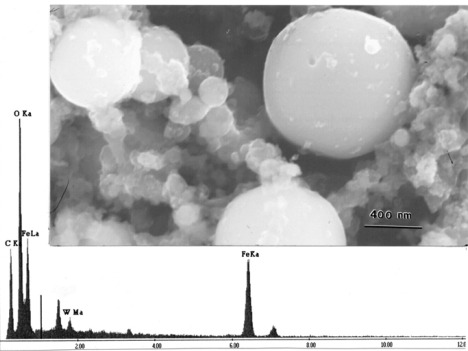
Single-filter sample particulates scraped from the filters into conductive carbon tape and observed in the FESEM. WA #1 WHA rod penetrator event image with broad-area EDS.

Figure 5 shows for comparison single (MCE) filter particulate collections for both WA #1. This SEM image illustrates notable clarity, in contrast to Figure 4 because the filters were pressed against sticky carbon SEM tabs which removed the particles and placed them on an electrically conducting support which largely eliminated charging of the specimens. The EDS insert in Figure 5 is essentially the same as those shown in Figures 3 and 4, with small variations in W concentrations and no Ni spectra.

**Figure 6.**
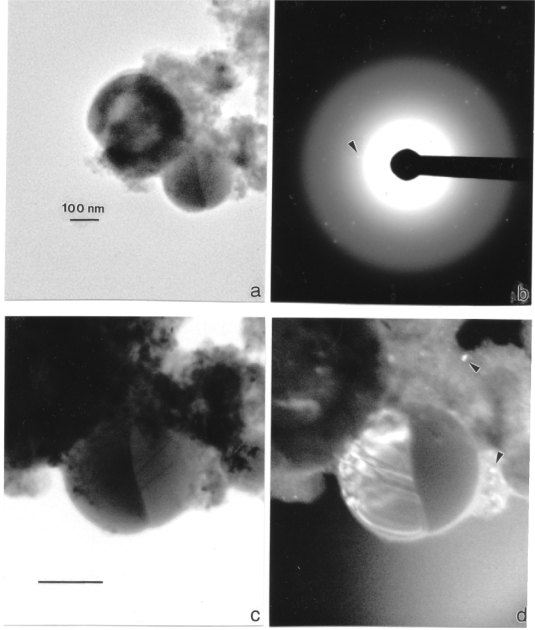
WA #1 WHA penetrator event aerosol particulates scraped from a single filter collector onto a grid sandwich and observed in the TEM. (a) Bright-field image of particulate aggregates. (b) Selected-area electron diffraction (SAED) pattern for aggregates in (a). Magnified bright-field image of (a). (d) Dark-field image of (c) using diffraction spot at arrow in (b). Nanoparticle images are indicated by the arrow. The magnification markers are designated by the marker in (a). The accelerating potential was 300 kV.

Figure 6 shows an analytical sequence for TEM observations of a small aggregate of particulates scrapped from an MCE filter for a WA #1 penetration event. Figure 6(a) illustrates a bright-field image of the aggregate while Figure 6(b) shows the corresponding selected-area electron diffraction (SAED) pattern. Only a few diffraction spots are observable above the diffuse background and the arrow indicates a specific diffraction spot for one of the larger (Fe) particles in Figure 6(a). This particle is shown magnified in Figure 6(c) while Figure 6(d) illustrates the corresponding dark-field image of Figure 6(c) utilizing the specific diffracting spot at the arrow in Figure 6(b). It can be noted that this particle is a bi-crystal with some microstructure visible in the left-hand crystal (bright). Several other much smaller (nano) crystal fragments are indicated at arrows in Figure 6(d). The smallest observable dark-field particle images in Figure 6(d) are observed to be ∼5 nm.

**Figure 7.**
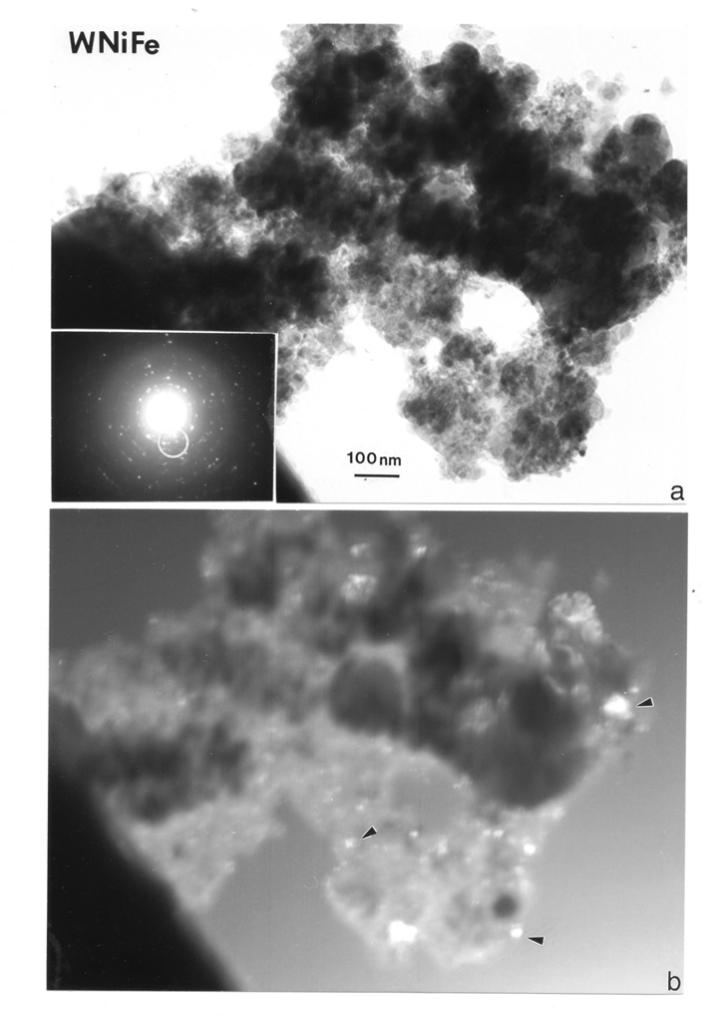
Single-filter collection sample for a WA #1 WHA rod penetration event scraped onto a grid sandwich and observed in the TEM. (a) Bright-field image of nanoparticulate aggregates. The SAED pattern insert illustrates broad crystallinity. (b) Dark-field image of (a) using electron diffraction spots with the aperture area shown circled in SAED pattern insert in (a). Nanocrystalline particulates are designated typically by arrows. (200 kV accelerating potential).

Figure 7 provides additional TEM bright-and dark-field image sequences showing the very small nanoparticulates to range from ∼10 to 50 nm. Numerous nanoparticulate aggregates are also visible in the dark-field images of Figures 7(b) for WA #1 and W-Ni-Co projectile firings, respectively. The SAED pattern inserts in Figures 7(a) show prominent arrays of diffraction spots from various crystalline particles, there is some diffuse diffraction which is less prominent than the SAED pattern insert in Figure 6(b). However this diffuse diffraction along with careful perusal of the bright-field images of particulates in Figures 6(a) and 7(a), suggests some organic (carbon) component of the aggregates, which is also reflected in the carbon peaks in all EDS inserts (Figures 3–5). At least some of this organic debris represents residual combustion material associated with the gun firing of the rod projectiles as noted above.

### 3.3. Cytotoxicity Assays

**Figure 8.**
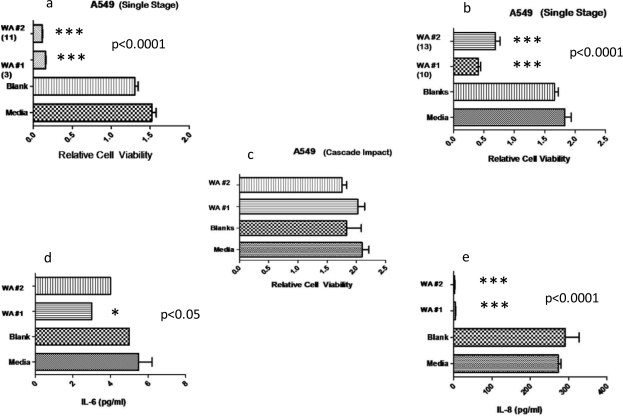
Comparative cytotoxicities (as relative A549 epithelial cell viability in filter-collected samples) and cytokine production. (a) and (b) Single filter exposure cell culture assays compared to media or untreated cell control and blank filter control for 48h exposure. Specific ballistic event numbers are shown for WA #1 and WA #2 penetration events. (c) Cascade impact stage 6 cell culture assays which show no effect. (d) and (e) show cytokine (IL 6 and IL 8) assays. Data is presented as the mean ± SEM of duplicate samples and is one of two representative experiments (*p ≤ 0.01).

Figure 8 shows the *in vitro* assays in A549 cell cultures for 48h for the six assay groups (see Table 2). Each experiment was done in duplicate (2 wells per treatment for each experiment) and each experiment was done twice. No significant reduction in A549 cell viability was associated with direct exposure to the stage 6 impactor collection (see Table 2, CI6-WA #1 and CI6-WA #2). In contrast, direct exposure of single stage filter samples from impacts of both WA #1 and 2 (SSF-WA #1 and SSF-WA #2) show very significant or highly cytotoxic responses. Respiratory epithelial cells such as the A549 lung model cells used in these assays have the ability to synthesize and release inflammatory cytokines such as interluken (IL)-6 and IL-8, as well as growth factors that modulate differentiation and inflammatory cells. Interleukin 6 (IL-6) and IL-8 are considered markers for inflammatory response both in the A549 cell culture models and in bronchial alveolar lavage fluids in animals [14–19], ideally providing a biomarker link between *in vitro* and *in-vivo* studies. Specifically, IL-6 has been associated with allergic responses involving asthma [16–19] while IL-8 has been linked with chronic obstructive pulmonary disease (COPD) [5,18–20]. The cells are killed so rapidly that there is no time to respond, as evident in the complete lack of IL production in Figure 8(d) and (e). The cells were killed so rapidly that only aggregates of particulates uptaken by the cells remained.

### 3.4. Comparative Cytoxicity of These and Other Nanoparticulate Species

Specimens collected on the single-stage filters represent the full spectrum aerosol specimens generated and collected. The stage 6 cascade impactor collections represent a subset of that present in the single-stage collections. Ideally, the latter collections contained a smaller overall size fraction of particulates, but both the single filter collections as well as the stage 6 impactor collections contained nanoparticulates and nanoparticulate aggregates as illustrated in figures 3–5. However, in contrast to the single filter cytotoxicities illustrated in Figure 8(a) and (b), there was no significant reduction in A549 cell viability for the stage impactor assays (CI6-WA #1 and #2) (Figure 8(c)). This indicates a sharp delineation in cytotoxicity with particulate and especially 6 nanoparticulate concentrations since, as indicated in Tables 1 and 2, the total and respirable fractions are roughly 4 times greater than the particulate concentrations on the stage 6 impactor filters, even though these filters contained significantly more particulates (particulate concentrations) than other impactor filters (Tables 1 and 2).

In contrast to other filter exposure cytotoxicity assays and standard *in vitro* cytotoxicity comparisons for a wide range of other nanoparticulate materials in culture (A549 cell exposure), the ballistic aerosol particulates are highly toxic [8,21]. Similar features have been observed for a wide range of nanoparticulates and nanoparticulate aggregates which also exhibit significant cytotoxicity [6].

### 3.5. Respiratory Effects of Nanoparticulates

There is a plethora of evidence over at least the past two decades that metal and metal oxide particles, especially nanoparticles are toxic, especially in the pulmonary system. Publications by Cugell *et al.* [22]; Sullivan *et al.* [23] and Buzea *et al.* [24] to name only a few attest to the health effects of Co, Ni, W, and Fe. These exposures to Co, Ni, and W can cause pulmonary fibrosis asthma, pulmonary eodema and pneumonia among other effects. Nickel and its compounds in particular have been demonstrated to cause nasal and lung cancers in the longer term for repeated exposure [23,24]. In addition, a wide range of metal oxides have also been demonstrated to be toxic or cytotoxic, especially iron oxide [24,25] as illustrated in the comparative cytotoxicity data in Figure 6.

Inhaled particulates (including coarse nanoparticles <1 μm) initially encounter the mucocillary clearance by cilia of the bronchial epithelial cells which moves the larger particles (>1 μm) towards the upper respiratory tract. However the truly nanoparticles (<1 μm) migrate to the alveoli where phagocytes and other cells with phagocytic abilities work to arrest them. However with phagocytic impairment, or for nanoparticles <100 nm which are not readily phagocitized, nanoparticles can accumulate and even aggregate to create oxidative stress and inflammation [26]. This can lead to various diseases while inflammation plays a major role in coronary heart disease and airway diseases such as asthma and chronic obstructive pulmonary disease (COPD). Severe inflammation is also associated with the onset of autoimmune disease. A prominent mechanism responsible for the variety of nanoparticle toxicities assumes a shift in the redox balance of the cells towards oxidation as a consequence of the formation of reactive oxygen species (ROS) which can, in the longer term, lead to DNA damage [24]. In addition to oxidative stress, some nanoparticles can enhance the expression of specific viral receptors and lead to severe inflammation when exposed to viral infections while other nanoparticles can decrease the expression of certain viral and bacterial receptors which lowers the resistance to some types of micro-organisms [27]. This phenomenon has been described previously for the *in vitro* assays for the collected aerosol particles on filters where IL-8 inhibition prevents the A549 epithelial cells from normal function. This would prevent the lungs from mounting an effective response to inhaled microbes.

Oberdörster, *et al.* [26] have demonstrated that aggregated nanoparticles are not as toxic as smaller concentrations of single particles. However, considerable aggregation observed in this study would suggest that in lung fluid, many of the aggregated particles, particularly those <100 nm, would disaggregate. Moreover, Murr *et al.* [25] have shown that a wide range of aggregated nanoparticulates are noticeably cytotoxic. Additionally high concentrations of aggregated nanoparticles can produce lung burden and pulmonary tissue damage depending upon the lung clearance rate [24]. Smaller nanoparticles (<30 nm) can emulate virus diameters and cross physiological barriers, entering the circulatory and lymphatic systems where they can translocate to various organs [28]. Inhaled nanoparticles <100 nm have also been shown to reach the brain via olfactory nerves as well as the blood-brain barriers [24,30]. Studies have suggested that high concentrations of metals such as Cu, Al, Fe, and others, together with oxidative stress, may initiate and even promote neurodegenerative diseases such as Parkinson’s and Alzheimer’s diseases [24].

Current research reviews indicate that with few exceptions, nanoparticles and nanoparticle aggregates are toxic (if not cytotoxic) to living organisms [24,25]. However, the relationship between nanoparticle exposure and immune response is not well known. The degree of toxicity for specific nanoparticles cannot be extrapolated from bulk properties, and materials which are nontoxic in bulk form may be toxic in nanoparticle or nanoparticle aggregate form [24].

## 4. Conclusions

As in the case for environmental particulate regimes, including the outdoor environment, nanoparticulates accounted for the most significant number concentration or particulate abundance of the ballistic aerosol, with mass abundances exceeding 150 mg/m^3^ (Table 3). This might be compared to mass concentrations of ∼90 mg/m^3^ for burning tire soot or natural gas combustion particulate mass concentrations of ∼0.01 mg/m^3^ as illustrated in the comparative cytotoxicity data in Figure 8(e)[30]. Correspondingly, these small mass abundance values represented the most cytotoxic aerosol category in contrast to larger concentrations of other soot species. In this work, ballistic aerosol composed largely of nanoparticulates averaging ∼10 nm (Figures 6–7) was shown to be so highly toxic to human epithelial cells over a short time (48 h) in culture that the cells died (Figure 8(c) and (d)) before they could produce any responsive cytokines (IL-6 or IL-8) (Figure 13(c) and (d)). Lower ballistic aerosol concentrations measured to be roughly ¼ of the topic concentrations showed no measurable cell (A549 human epithelial) death after 48 h in culture. While the concentration cut off and exposure time in culture to affect significant epithelial cell death was not determined, the aerosol characterization and cytotoxicity assays provide compelling evidence for significant health hazards “for exposure, inhalation, and respiration of aerosolized metals” as earlier suggested by Gold *et al.* [1] for military crew compartment armor steel perforation by a WHA-KE penetrator of the type studied in this work.

Although the nanoparticulate species or stoichiometries contributing significantly to epithelial cell death (Figures 8(a), (b), (c), and (d)) are not fully characterized, considerable quantities (concentrations) of iron oxides and other metal or metal oxide nanoparticles contribute significantly to the ballistic aerosol and these have already been demonstrated to be highly cytotoxic as illustrated in Figure 8(f). It is also not known how these various nanoparticle species contribute synergistically to the highly toxic behavior of the ballistic aerosol. Despite these shortcomings, this research suggests that there is the potential for severe or chronic respiratory health effects associated with even short-term exposure to ballistic aerosol at high concentrations associated with KE penetrator perforation of crew compartments in a variety of armored vehicles.

## Figures and Tables

**Table 1.** Composition of M2 propellant.

**M2 Composition**	**%**
Nitrocellulose	77.45
Nitroglycerin	19.50
Ba (NO_3_)_2_	1.40
KNO_3_	0.75
Ethyl Centralite	0.60
Graphite	0.30

**Table 2.** Direct Contact Cytotoxicity Assay Groups.

**Name**	**Description**
SSF-WA #1	Single Stage Filter Collections for WA #1 impacting target array
SSF-WA #2	Single Stage Filter Collections for WA #2 impacting target array
CI6-WA #1	Cascade Impactor Stage 6 Specimens for WA #1 impacting array
CI6-WA #2	Cascade Impactor Stage 6 Specimens for WA #1 impacting array
Media	Negative Control-Untreated cells
Blank	Cells exposed to a single stage filter on which nothing has been collected

**Table 3.** Particulate collection for WA #1 impact experiment.

**Stage**	**Cut Point (microns)**	**Total Particulate Weight (mg)**	**Total Particulate Concentration (mg/m^3^)**	**Iron Conc. (mg/m^3^)**	**Nickel Conc. (mg/m^3^)**	**Tungsten Conc. (mg/m^3^)**
1	21	0.432	17	14.8	0.286	1.92
2	15	0.052	2	4.51	0.134	1.36
3	10	0.000	0	3.52	ND	0.217
4	6	0.049	2	7.31	0.118	0.318
5	3.5	0.798	31	21.0	0.312	0.736
6	2	0.953	38	20.9	0.290	0.781
7	0.9	0.677	27	9.97	0.142	0.625
8	0.5	0.204	8	2.25	ND	0.401
Backup Filter	0.25	0.878	35	4.89	ND	2.59
Total			160	89.2	1.28	8.95
Respirable			106	65.0	0.862	3.08

ND—Concentration below instrument detection limit.

**Table 4.** Particle collection for 91% W-6%Ni, 3% Co (WHA) penetrator impact experiment.

**Stage**	**Cut Point (μm)**	**Total Particulate Weight (mg)**	**Total Particulate Concentration (mg/m^3^)**	**Cobalt Conc. (mg/m^3^)**	**Iron Conc. (mg/m^3^)**	**Nickel Conc. (mg/m^3^)**	**Tungsten Conc. (mg/m^3^)**
1	21	0.216	6	ND	5.85	0.114	0.274
2	15	0.000	0	ND	1.70	ND	0.102
3	10	0.254	7	ND	1.87	ND	0.106
4	6	0.030	1	ND	3.96	0.076	0.185
5	3.5	0.841	22	0.088	13.70	0.208	0.535
6	2	0.981	26	0.068	12.40	0.174	0.580
7	0.9	0.592	16	0.184*	5.17	0.079*	0.125*
8	0.5	0.310	8	0.075	3.07	ND	0.417
Backup Filter	0.25	1.131	30	ND	4.20	0.090	1.380
Total			116	0.415	51.90	0.741	3.700
Respirable			80	0.415	40.20	0.537	1.950

ND—Concentration below instrument detection limit.
